# Developmental Sex Differences in the Metabolism of Cardiolipin in Mouse Cerebral Cortex Mitochondria

**DOI:** 10.1038/srep43878

**Published:** 2017-03-06

**Authors:** Estefanía Acaz-Fonseca, Ana Ortiz-Rodriguez, Ana B. Lopez-Rodriguez, Luis M. Garcia-Segura, Mariana Astiz

**Affiliations:** 1 Instituto Cajal-CSIC. Avenida Doctor Arce 37, 28002 Madrid, Spain; 2 Biochemistry Research Institute of La Plata (INIBIOLP), CONICET. 60 and 120, 1900, La Plata, Argentina

## Abstract

Cardiolipin (CL) is a mitochondrial-specific phospholipid. CL content and acyl chain composition are crucial for energy production. Given that estradiol induces CL synthesis in neurons, we aimed to assess CL metabolism in the cerebral cortex (CC) of male and female mice during early postnatal life, when sex steroids induce sex-dimorphic maturation of the brain. Despite the fact that total amount of CL was similar, its fatty acid composition differed between males and females at birth. In males, CL was more mature (lower saturation ratio) and the expression of the enzymes involved in synthetic and remodeling pathways was higher, compared to females. Importantly, the sex differences found in CL metabolism were due to the testosterone peak that male mice experience perinatally. These changes were associated with a higher expression of UCP-2 and its activators in the CC of males. Overall, our results suggest that the perinatal testosterone surge in male mice regulates CL biosynthesis and remodeling in the CC, inducing a sex-dimorphic fatty acid composition. In male’s CC, CL is more susceptible to peroxidation, likely explaining the testosterone-dependent induction of neuroprotective molecules such as UCP-2. These differences may account for the sex-dependent mitochondrial susceptibility after perinatal hypoxia/ischemia.

Cardiolipin (CL) is a unique phospholipid that is almost exclusively localized at the inner mitochondrial membrane (IMM). CL is structurally unique from other glycerophospholipids since it contains four, rather than two, fatty acyl side chains. CL plays a relevant role in mitochondrial function, which is now appreciated to be multifaceted. CL content and fatty acid (FA) composition determine membrane fluidity and, therefore, affect the assembly of protein complexes^1^,^2^. CL contributes to the stabilization of the oxidative phosphorylation (OXPHOS) machinery and uncoupling proteins (such as UCP-2), increasing the efficiency of ATP synthesis^3^,^4^,^5^,^6^,^7^,^8^. Moreover, CL stabilizes mitochondrial outer membrane translocators (translocase of the outer membrane, TOM and sorting and assembly machinery, SAM)^9^, regulates apoptosis by serving as a recruitment platform for caspase 8 downstream of Fas receptor signaling and is involved in cytochrome C release upstream of caspase 3^10^,^11^. CL is also involved in mitochondrial fission/fusion processes^12^,^13^, as well as in the initiation of mitophagy^14^,^15^. Thus, alterations in CL structure, content, and acyl chain composition have been associated with mitochondrial dysfunction in multiple tissues in several physiopathological conditions^16^.

CL synthesis occurs entirely in the mitochondria. Phosphatidylglycerolphosphate synthase (PGS-1) catalyzes the first step in CL biosynthesis, transforming cytidinediphosphate (CDP)-diacylglycerol (CDP-DAG) in the short-lived phosphatidylglycerolphosphate (PGP), which is then desphosphorylated. CL synthase (CLS) generates immature CL from phosphatidylglycerol and another molecule of CDP-DAG. Immature CL is characterized by a random assortment of its acyl chains, which are mainly saturated and variable in length. An acyl chain remodeling process, which is typically defined by the incorporation of longer and unsaturated fatty acyl chains, is responsible for the molecular composition of mature cardiolipin^17^. This remodeling process is initiated by a phospholipase, in mammals the calcium-independent iPLA2-γ^18^, which removes an acyl chain from cardiolipin, generating monolysocardiolipin (MLCL). Taffazin (Taz) then reacylates MLCL, by exchanging an acyl chain from another phospholipid, preferentially phosphatidylcholine (PC) or phosphatidylethanolamine (PE). Alternatively, lysocardiolipin acyltransferase-1 (LCLAT-1) utilizes an acyl-CoA as a donor for reacylating MLCL^19^. This remodeling process is highly tissue-specific, indeed, cardiac CL from adult mice is almost exclusively composed by linoleic acid (18:2), while adult brain CL is enriched in a variety of long and highly unsaturated FAs^20^,^21^.

Interestingly, our current knowledge in CL metabolism comes from studies in adult tissues^22^; however, little is known about the role of this phospholipid during brain development. Few years ago, was demonstrated that the large-scale reorganization of neuronal circuits after birth is accompanied by intense mitochondrial activity, high expression of uncoupling proteins and profound alterations in CL content and molecular species distribution^23^,^24^,^25^,^26^. During embryonic development, CL contains relatively high amounts of short aliphatic chains (palmitoleic and oleic acids). In contrast, after birth CL becomes enriched in long chain polyunsaturated FAs (PUFAs), such as arachidonic and docosahexanoic acids^25^. This rapid CL remodeling may affect the susceptibility to peroxidation, the physical properties of the IMM, the stability and activity of integral protein complexes, likely playing a role in brain development.

Around birth, testosterone produced by the developing testes in male mice, enters the brain and is aromatized to estradiol and plays an essential role in brain sex differentiation^27^,^28^. Since the effect of estradiol inducing CL synthesis has been shown in previous studies in cultured neurons^29^, we hypothesized that CL metabolism and structure are influenced by the testosterone surge during the perinatal period. To test this hypothesis we have studied CL levels and CL fatty acid composition in mitochondria from cerebral cortex (CC) of newborn male and female mice, as well as the impact of the hormonal status on the CL biosynthetic pathways.

## Results

### Sexual dimorphism in CL levels and CL fatty acid composition in mitochondria from mice CC

**Figure 1:**
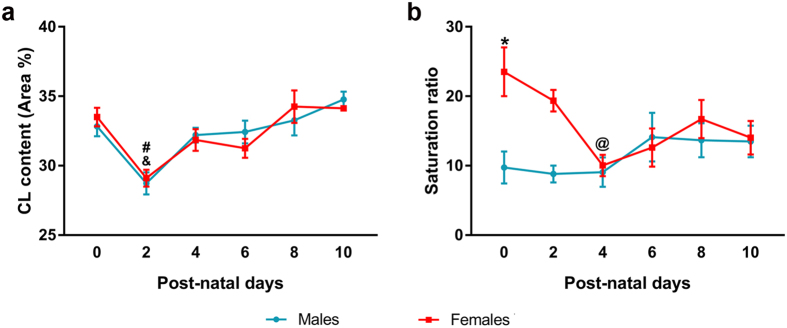
CL content and CL saturation ratio in mitochondria from the cerebral cortex of postnatal male and female mice. (**a**) CL content in mitochondria from cerebral cortex of male and female mouse pups. (**b**) CL composition in mitochondria from the cerebral cortex of male and female mouse pups, expressed as the saturation ratio (∑ Saturated (16:0 + 18:0)/∑ Polyunsaturated (18:2 + 18:3 + 20:4 + 22:5 + 22:6)). Sample size N ≥ 4. *Significant differences (p < 0.05) vs. Females for each time point. ^&^Significant differences (p < 0.05) vs. Females at the previous time point. ^#^Significant differences (p < 0.05) vs. Males at the previous time point. ^@^Significant difference (p < 0.05) vs. Females at PND 0.

To assess CL content and composition, mitochondria were extracted from the CC of male and female mice at different days after birth (Fig. 1). CL content was not affected by the sex of the animals (F = 0.002, p = 0.962), but was significantly affected by their age (F = 12.183, p = 0.000). Given that there was no interaction between these two factors (F = 0.556, p = 0.733), the effect of age was analyzed for each sex independently by one-way ANOVA. There was a significant effect of age in both sexes (F = 6.098, p = 0.002 in males and F = 6.627, p = 0.002 in females), CL content decreased significantly at PND 2 and restored up by PND 10 (Fig. 1a).

CL fatty acid composition was assessed by liquid-gas chromatography and a saturation ratio was calculated (Saturation ratio = ∑ Saturated/∑ Polyunsaturated, Fig. 1b). Two-way ANOVA revealed a statistically significant effect of sex (F = 10.297, p = 0.003), but not of age (F = 2.214, p = 0.072) on the distribution of the saturation ratio. A significant interaction between these two factors was detected (F = 3.102, p = 0.019). According to the *post*-*hoc* test, CL from females showed a higher proportion of saturated FAs than males at birth. By PND 4, the saturation ratio significantly decreased in females and remained similar to that of males until PND 10 (Fig. 1b).

### Androgenization of female pups abolishes sex differences in CL saturation ratio

**Figure 2:**
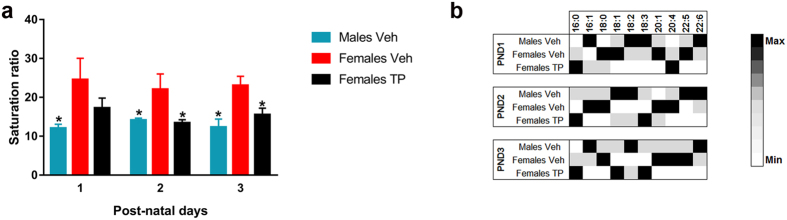
Androgenization of female pups abolishes sex differences in CL saturation ratio in mitochondria from the developing cerebral cortex. (**a**) CL composition in the cerebral cortex of male and female mouse pups treated with vehicle and females treated with TP at PND 1, 2 and 3, expressed as the saturation ratio (∑ Saturated (16:0 + 18:0)/∑ Polyunsaturated (18:2 + 18:3 + 20:4 + 22:5 + 22:6)). (**b**) Relative amount of CL species in the cerebral cortex of Males Veh, Females Veh and Females TP at PND 1–3. Sample size N ≥ 3. *Significant differences (p < 0.05) vs. Females Veh for each time point.

Only male mice experience an increase in testosterone plasma levels around birth, which defines a critical hormonal status for the sexual dimorphic development of the brain. In order to assess the influence of the hormonal status in CL composition, we emulated the physiologic perinatal testosterone peak in females, through the injection of testosterone propionate (TP) at PND 0. CL-fatty acid composition in males (Males Veh), females (Females Veh) and TP- injected females (Females TP) was assessed at PND 1, 2 and 3 (Fig. 2). Regarding the saturation ratio of CL (Fig. 2a), two-way ANOVA revealed a significant global effect of the hormonal status (F = 13.001, p = 0.000), independently of the age (F = 27.470, p = 0.686). Given that there was no significant interaction between the hormonal status and age, the differences between groups were analyzed for each time point. Females Veh showed a significantly higher saturation ratio than Males Veh. However, the saturation ratio in Females TP was similar to that of Males Veh, while significantly different from that of Females Veh at PND 2 and PND 3 (Fig. 2a). Interestingly, the effect of testosterone on the saturation ratio could not be attributed to changes in any specific FAs, but rather to a global effect on the proportion of most of them (Fig. 2b). Indeed, the specific CL species enriched in TP females were different from those in males.

### Effect of neonatal testosterone on CL biosynthetic pathways

**Figure 3:**
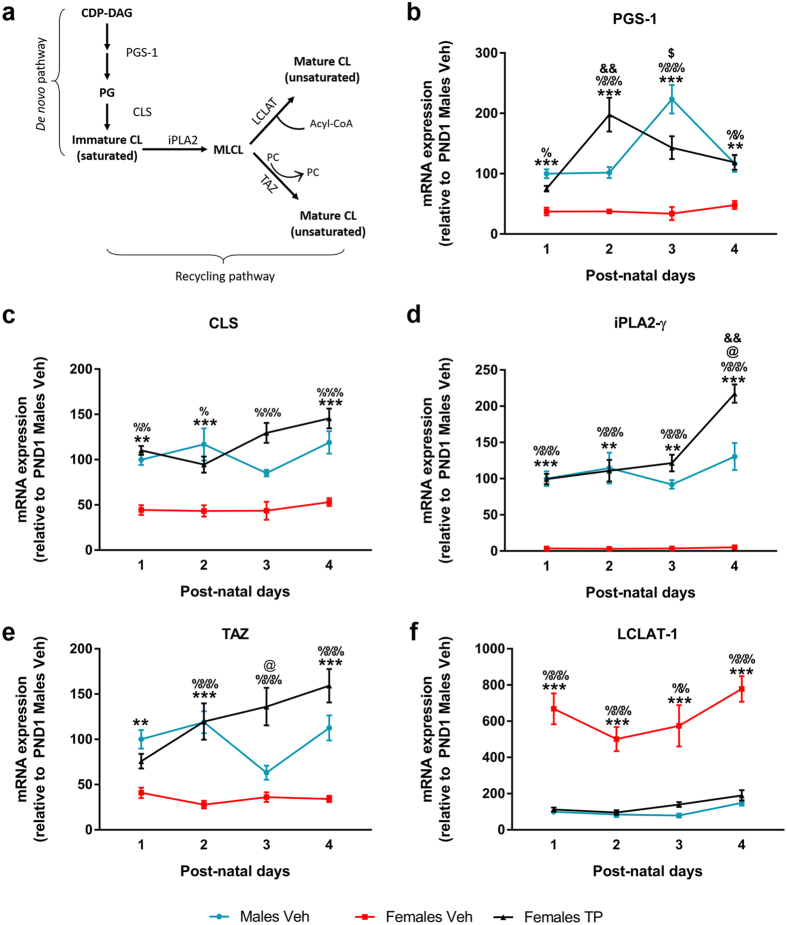
Sex differences and effect of neonatal testosterone on CL biosynthetic pathways. (**a**) Scheme of CL synthesis. The *de novo* synthesis is catalized by Phosphatidylglycerolphosphate synthase (PGS-1) which transforms cytidinediphosphate CDP-diacylglycerol (CDP-DAG) into phosphatidylglycerolphosphate (PGP), which is later desphosphorylated. CL synthase (CLS) forms immature CL from phosphatidylglycerol and another molecule of CDP-DAG. The remodeling of CL is initiated by the calcium-independent phospholipase A2 gamma (iPLA2-γ), which removes the acyl chains from CL and generates the intermediate monolysocardiolipin (MLCL). Taffazin (Taz), or alternatively lysocardiolipin acyltransferase-1 (LCLAT-1), reacylate MLCL to CL. (**b–f**) mRNA expression of the main enzymes involved in CL synthesis: PGS-1 (**b**), CLS (**c**), iPLA2-γ (**d**), TAZ (**e**) and LCLAT-1 (**f**). Sample size N ≥ 4. **^,^ ***Significant differences (p < 0.01 and p < 0.001) between Males Veh and Females Veh at each time point. ^%, %%,^^%%%^Significant differences (p < 0.05, p < 0.01 and p < 0.001) between Females Veh and Females TP at each time point. ^$^Significant difference (p < 0.05) vs. Males Veh PND 1. ^&&^Significant differences (p < 0.01) vs. Females TP PND 1. ^@^Significant (p < 0.05) differences between Females TP and Males Veh.

As mentioned before, the CL fatty acid composition strongly depends on the activity of both the *de novo* and the remodeling synthetic pathways (represented in Fig. 3a). Thus, in order to assess whether the influence of the hormonal status on the saturation ratio was due to an effect on the expression of the enzymes involved in CL synthesis, we quantified the mRNA expression of enzymes from both the *de novo* pathway (Fig. 3b and c) and the remodeling pathway (Fig. 3d,e and f).

Phosphatidylglycerolphosphate synthase (PGS-1) catalyzes the first and committed step in cardiolipin *de novo* biosynthesis. PGS-1 mRNA expression (Fig. 3b) was significantly influenced by both, the hormonal status (F = 107.586, p = 0.000) and the age (F = 4.485, p = 0.007). Besides, these two factors showed a statistically significant interaction (F = 5.636, p = 0.000). Females Veh displayed a constant expression of PGS-1 along time, while Males Veh showed a peak of PGS-1 expression at PND 3 and Females TP at PND 2. PGS-1 expression levels were significantly lower in Females Veh compared to Males Veh and to Females TP at every time point studied.

Cardiolipin synthase (CLS) forms CL by condensing phosphatidylglycerol (PG) and a molecule of CDP-DAG. mRNA levels of CLS (Fig. 3c) were also affected by the hormonal status (F = 77.839, p = 0.000) and age (F = 4.006, p = 0.012), showing a significant interaction (F = 2.682, p = 0.024). The expression of CLS in Females Veh is lower than in Males Veh at every time point, except at PND 3. TP treatment after birth significantly increased CLS mRNA expression in female pups, reaching similar levels of expression to those found in Males Veh.

The remodeling process of CL is initiated by the calcium-independent phospholipase A2γ (iPLA2-γ). Later, a reacylation step is catalyzed by Taffazin (Taz) by exchanging acyl chains from other phospholipids or, alternatively, by lysocardiolipin acyltransferase-1 (LCLAT-1), which uses an acyl-CoA as a donor for the reacylation (Fig. 3a).

The transcription of iPLA2-γ (Fig. 3d) was significantly affected by age (F = 7.250, p = 0.000) and the hormonal status (F = 108.230, p = 0.000) of the animals; however the interaction between these two factors was not significant (F = 1.251, p = 0.296). Hence, in order to assess the effect of each factor independently, data were split and analyzed by one-way ANOVA. Females Veh showed significantly less iPLA2-γ mRNA expression than Males Veh and Females TP at each time point. Moreover, Females TP was the only group in which iPLA2-γ mRNA expression was dependent on the age of the animals. At PND 4, iPLA2-γ transcription significantly increased in Females TP (compared to PND 1), but the transcription of this gene in the rest of groups remained stable along time. This fact drove to the appearance of a significant difference between Females TP and Males Veh at PND 4 (Fig. 3d).

TAZ mRNA expression (Fig. 3e), analysed by two-way ANOVA, revealed a significant effect of the hormonal status (F = 101.831, p = 0.000) but not of the age (F = 1.948, p = 0.134). A significant interaction (F = 4.471, p = 0.001) between these factors was detected. Females Veh showed lower expression levels of TAZ mRNA than Males Veh at all ages except for PND 3, and than Females TP at all ages.

LCLAT-1 mRNA expression (Fig. 3f) was significantly influenced by both the hormonal status (F = 131.244, P = 0.000) and the age of the animals (F = 13.473, p = 0.000), but these factors did not show a significant interaction (F = 2.094, p = 0.07). In order to assess the effect of each factor, data were split and the differences analyzed by one-way ANOVA. LCLAT-1 mRNA expression was significantly higher in Females Veh than in Males Veh and than in Females TP, at every time point. In addition, the age of the animals had a significant impact on the transcription of LCLAT-1 in Males Veh (F = 6.810, p = 0.004) and Females TP (F = 5.922, p = 0.007), but not in Females Veh (F = 2.604, p = 0.080).

### Effect of neonatal testosterone on fatty-acid desaturases expression

**Figure 4:**
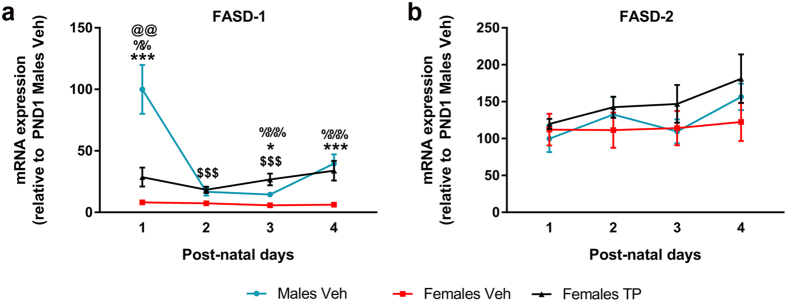
Sex differences and effect of neonatal testosterone on desaturases. mRNA expression of FADS-1 (Fatty acid desaturase-1) (**a**) and FADS-2 (Fatty acid desaturase-2) (**b**). Sample size N ≥ 4. *^,^ ***Significant differences (p < 0.05 and p < 0.001) between Males Veh and Females Veh at each time point. ^%%, %%%^Significant differences (p < 0.01 and p < 0.001) between Females Veh and Females TP at each time point. ^$$$^Significant difference (p < 0.001) vs. Males Veh PND 1. ^@@^Significant (p < 0.01) differences between Females TP and Males Veh.

To assess the possible effect of the hormonal status on the availability of unsaturated FAs, the expression of the rate-limiting enzymes in their byosinthesis was quantified. FADS-1 (Fatty acid desaturase-1) mRNA expression (Fig. 4a) was significantly influenced by both the hormonal status (F = 85.665, P = 0.000) and the age of the animals (F = 10.736, p = 0.000), and a significant interaction was detected between the two factors (F = 6.396, p = 0.000). Females Veh showed lower expression levels of FADS-1 than Males Veh and Females TP at all ages (Fig. 4a).

FADS-2 (fatty acid desaturase-2) mRNA expression (Fig. 4b) was neither influenced by the hormonal status (F = 2.877, P = 0.065), nor by the age of the animals (F = 1.534, p = 0.217); these factors did not show a significant interaction (F = 0.331, p = 0.917) (Fig. 4b).

### Effect of neonatal testosterone on UCP2 and PPAR-α expression

**Figure 5:**
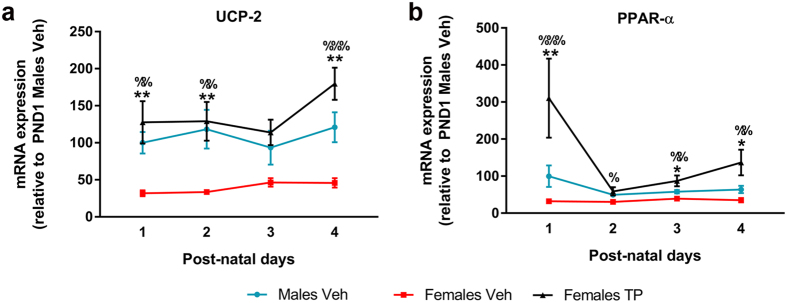
Sex differences and effect of neonatal testosterone on UCP2 and PPAR-α expression. mRNA expression of UCP2 (**a**) and PPAR-α (**b**). Sample size N ≥ 4. *^,^ **Significant differences (p < 0.05 and p < 0.01) between Males Veh and Females Veh at each time point. ^%, %%, %%%^Significant differences (p < 0.05, p < 0.01 and p < 0.001) between Females Veh and Females TP at each time point.

To investigate a possible impact of the sexual dimporphic composition of CL on oxidative phosphorylation efficiency, we assessed the mRNA expression of UCP-2 (one of the main UCPs isoforms in the brain) and its activator peroxisome proliferator-activated receptor alpha (PPAR-α)^30^,^31^ in the androgenization model (Fig. 5). UCP-2 mRNA expression (Fig. 5a) was dependent on the hormonal status of the animals (F = 48.060, p = 0.000), but not on their age (F = 1.645, p = 0.191). These two factors did not show a significant interaction (F = 1.082, p = 0.385); therefore, the effect of the hormonal status was assessed at each time point by one-way ANOVA. UCP-2 expression was significantly lower in Females Veh than in Males Veh and Females TP except for pups at PND 3 (where the variable was not dependent on the hormonal status, F = 3.693 and p = 0.056). According to the two-way ANOVA, the transcription of PPAR-α (Fig. 5b) was significantly affected by the hormonal status (F = 50.304, p = 0.000) and by age (F = 4.634, p = 0.006). Given that there was no significant interaction between these factors (F = 1.151, p = 0.347), the effect of the hormonal status was assessed at each time point by one-way ANOVA. Females Veh showed significantly lower levels of PPAR-α expression than Males Veh, and also than Females TP except at PND 2. The expression of PPAR-α was not affected by postnatal age.

**Figure 6:**
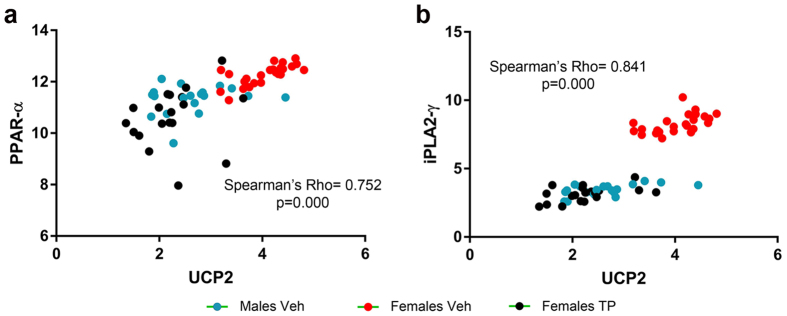
Correlation of UCP2 mRNA expression with PPAR-α(A) and iPLA2-γ(B) mRNA levels. Number of XY pairs = 62 for global analyses, 19 for Males Veh and Females TP groups and 24 for Females Veh group.

Moreover, we evaluated a possible correlation between PPAR-α and UCP-2 mRNA expression by a Spearman test. For each sample, paired ΔCt values for UCP-2 and PPAR-α were plotted (Fig. 6a). Given that changes in PPAR-α mRNA expression are translated into changes in its protein levels and activity^32^, this correlation was performed as an approach to figure out whether PPAR-α protein could increase UCP-2 transcription in our model. The Spearman’s test revealed a statistically significant (Rho = 0.752, p = 0.0000) correlation between the transcription of PPAR-α and UCP-2 (Fig. 6a).

UCP-2 mRNA expression (Fig. 5a) and CL saturation ratio (Fig. 2a) were both influenced by the sex of the animals and by the hormonal status. Since free FAs, mainly produced by iPLA2-γ, are able to activate UCP-2^33^, we evaluated by an indirect approach whether the transcription of UCP-2 and iPLA2-γ may be related. Again, for each sample, paired ΔCt values for UCP-2 and iPLA2-γ were plotted and a global Spearman’s correlation was performed (Fig. 6b). The analysis revealed a significant correlation (Rho = 0.841, p = 0.000) between the mRNA expression of UCP2 and iPLA2-γ. These data were clearly arranged into two different populations, one of them containing the information from Females Veh (red spots) and the other one formed by the intermingled data from Males Veh (blue) and Females TP (black). Given this obvious arrangement of the data by the hormonal status of the animals, a Spearman’s test was applied for each experimental group in order to discard a possible spurious correlation in the global analysis. A significant correlation between the transcription of UCP-2 and iPLA2-γ was found in Males Veh (Rho = 0.553, p < 0. 014), Females Veh (Rho = 0.643, p < 0.001) and Females TP (Rho = 0.505, p < 0.027).

## Discussion

During embryonic development in mammals, the pituitary of male fetuses releases gonadotropins, which induce a sudden testosterone production by their testis^34^,^35^. Plasmatic testosterone reaches the brain^36^, where is transformed into estradiol or DHT by the enzymes aromatase and 5α-reductase, respectively^27^. Both metabolites are involved in organizational actions of testosterone in the nervous system of males, contributing to long-term sexual dimorphisms at the molecular, anatomical and behavioral levels^28^,^37^,^38^,^39^,^40^.

Despite the difference in the hormonal status between male and female pups, CL content in the CC was similar for both sexes during the first 10 days of post-natal life. Interestingly, CL content decreased significantly at PND 2. In experimental models of hypoxia/reperfusion, CL and cytochrome c are released from mitochondria after re-oxygenation in a ROS-dependent way^41^. The transient decay in cortical CL, already observed^25^, may be a consequence of the moderate hypoxia/reperfusion process associated with vaginal birth^42^, which also includes a moderate ATP depletion in the nervous tissue^43^,^44^.

Unlike total content, the fatty acid composition of CL was different between male and female pups at birth. The saturation ratio (∑ (16:0 + 18:0)/∑ (18:2 + 18:3 + 20:4 + 22:5 + 22:6)) reflects the maturation state of CL; thus, CL in the CC of females is more immature than in males at PND 0, but it is progressively recycled to reach the same maturation level than in males by PND 4. Given that CL composition experiences a progressive exchange of saturated FAs by polyunsaturated FAs (PUFAs) with age^25^,^45^, we speculate that CL also suffers an intense remodeling and maturation process after birth, and that it is slower or delayed in females than in males.

As stated above, CL strongly binds to protein complexes at the IMM^46^,^47^. This binding confers stability to both the protein supercomplexes and CL and slows down CL turnover^48^. Taking into consideration that most of the studies regarding CL metabolism have been obtained from fully mature tissues, the dynamic character of CL composition in the brain during the first days of life could be explained by the extremely active process of mitochondrial turnover that takes place during this period^26^.

In order to elucidate whether the perinatal testosterone surge has an impact on the sexual differences in CL molecular species we used a classical model of neonatal female androgenization^49^,^50^,^51^. Our findings indicate that the fatty acid composition of cortical CL is regulated by testosterone, explaining at least in part, its sex-dimorphic pattern during early postnatal life. Testosterone propionate treatment induced an enrichment of PUFAs in CL from female mice, achieving an overall saturation ratio similar to males, but through the increase of different CL species. A similar effect of testosterone on phospholipid FA composition was described before^52^,^53^. Further experiments are necessary to find other factors, apart from testosterone, regulating the specific enrichment of CL species in males. Among these factors, cell-autonomous effects of sex chromosomes outside of the gonads have been implicated in the generation of sex differences in different brain parameters^54^,^55^ and are, therefore, a potential mechanism to induce developmental sex differences in the enrichment of different CL species in the cerebral cortex (see below).

Additionally, in order to find out the molecular pathway responsible for this sexual dimorphism, we analyzed whether the effects of testosterone on CL fatty acid composition were due to the regulation of the enzymes involved in CL *de novo* synthesis or in CL remodeling. The mRNA expression of all the enzymes analyzed remained stable from PND 0 to PND 4, as had been previously shown^25^. Strikingly, all these enzymes showed a clear sex-dimorphic expression pattern, which was dependent on perinatal testosterone. PGS-1 and CLS, involved in CL *de novo* synthesis, were more abundantly transcribed in males and androgenized females than in females. Despite the higher ratio of *de novo* synthesis found in males (determined by the mRNA expression of CLS and PGS-1), the total amount of CL was similar in both sexes, likely due to the almost 100-fold higher expression of iPLA2-γ found in males compared to females.

Among the remodeling enzymes, testosterone induced TAZ mRNA expression, explaining the lower saturation ratio of CL in males compared to females. However, LCLAT-1 expression was unexpectedly higher in females and was repressed by testosterone, which goes against the observed sex difference in saturation ratio. The specificity of the recycling enzymes for certain FAs is still under debate. In fact, it was recently shown that the remodeling specificities are driven by the physical properties of the lipids rather than by the enzymes themselves^22^. Therefore, the observed sex difference in saturation ratio may relay on the availability of PUFAs for the reacylation process. In fact, while the expression FADS-1, one of the rate-limiting enzymes in long-chain PUFAs byosinthesis, was higher in males and was induced by testosterone, the expression of FADS-2 was similar in all the groups.

Overall, our findings point out the idea that perinatal testosterone enhances CL recycling in the mouse CC, resulting in a sex-specific saturation ratio of this essential mitochondrial phospholipid. Further research needs to be done in order to elucidate the underlying molecular mechanisms mediating the effects of the perinatal testosterone surge on CL composition. Nevertheless, a preliminary *in silico* analysis was performed to predict the presence of different transcription factor binding sites (TFBSs) in the promoters of the genes involved in CL metabolism. By combining the results provided by different search tools (PROMO v3.0.2^56^, TFIS^57^ and GPMiner^58^) we found binding sites for estrogen receptors in the promoters of PGS-1, CLS, iPLA2-γ and TAZ. However, the androgen receptor binding sites seem to be absent from the promoters of all these genes. This suggests that the effect of testosterone is mediated by its metabolite estradiol, synthesized in the brain by the enzyme aromatase. Although perinatal testosterone had a clear effect on LCLAT-1 mRNA expression, no ER or AR binding sites were predicted in the LCLAT-1 promoter. It is important to recall, however, that estrogens are able to modulate transcription by regultating other transcription factors^59^. For instance, estrogens activate AP-1^60^, which was predicted to have a binding site in LCLAT-1 promoter. Furthermore, the search tools also predicted that binding sites for SRY might be present in the promoters of CLS, iPLA2-γ and LCLAT-1. SRY, also known as “the sex determining factor”, is a transcription factor encoded in the Y chromosome and responsible of the masculinization of embryonic gonads during development^61^,^62^,^63^. Since SRY gene is expressed in the brain^64^, a direct contribution of sex chromosomes in the generation of sex differences is possible.

Given the crucial role of CL in mitochondrial function, the hormone-induced sexual differences in the metabolism of CL shown here, may set a possible explanation for the well-documented sexual dimorphisms in the outcome of perinatal hypoxia^65^. Males show higher rates of neuronal death and deeper cognitive impairments than females when exposed to the same hypoxic/ischemic (HI) injury^66^. Cell death in these models is a consequence of the reoxygenation process that follows ischemia^67^, thus, sex differences in mitochondrial function and membrane composition may underlie these differential outcome. Indeed, male pups present a deeper deficit in electron chain transport and a weaker anti-oxidant system than females after HI^68^,^69^,^70^. From our results, CL molecular species in males are enriched in PUFAs and therefore more sensitive to oxidative stress^71^. In addition, it was demonstrated that the nature of the fatty acid chains of CL influences both, the conformational and ion transport properties of neuronal UCP-2^8^. UCP-2 is considered to be a neuroprotective factor^72^,^73^ that is overexpressed during oxidative stress or after brain injuries^74^,^75^,^76^, contributing to decrease the production of ROS in the mitochondria^77^. Interestingly, UCP-2 expression increases in the mice brain after vaginal birth^23^, supporting the idea of vaginal birth as a mild hypoxic insult for the brain. In our experiments, UCP-2 expression was higher in males than in females as a consequence of the perinatal testosterone peak, possibly meaning that the nervous tissue from males needs a higher protection against oxidative stress than in females. On the other hand, PPAR-α activates UCP-2 transcription^78^,^79^,^80^, thus, the significant positive correlation between PPAR-α and UCP-2 mRNA expression, supports the possibility that the testosterone-induced PPAR-α expression/activity contributes to increase UCP-2 transcription in male pups. Furthermore, the increased expression of iPLA2-γ in the male CC may induce the activity of UCP-2 by increasing the amount of free FAs (FFAs)^81^,^82^. FFAs are also able to activate PPAR-α^83^,^84^, which in turn would reinforce the transcription of UCP-2.

In summary the present work demonstrates that the perinatal testosterone surge regulates CL biosynthesis in the CC, by increasing its turnover and maturation in males, relative to females. Consequently, CL from males is enriched in PUFAs, being more susceptible to peroxidation and oxidative stress, which may be in part compensated by a higher uncoupling of the electron transport chain. Although our findings would be strengthened by assessing other brain regions at this specific developmental stage, the physiological sex differences found in the CC could partially explain the sex-dependent mitochondrial dysfunctions observed after perinatal hypoxia/ischemia events; however, further studies using a perinatal hypoxia model would be necessary to contrast this hypothesis.

## Methods

### Drugs

All drugs and chemicals were obtained from Sigma Chemical Co., St. Louis, MO, USA. Organic solvents (Carlo Erba, Milan, Italy) and phospholipid standards were obtained from Avanti Polar Lipids Inc. (Alabama, USA).

### Mice

Wild-type C57Bl/6J mice were provided by the animal facility of the University of La Plata (UNLP). All the procedures were revised by the Institutional Animal Care and Use Committee (IACUC) of the Medicine School, UNLP. The approved protocol (# P01-01-2015) is in agreement with local guidelines for vertebrate animal welfare as well as with USPHS and/or European Union policy. Adult virgin female mice (8–10 weeks old) were group-housed (5 per cage) to coordinate their estrous cycles in a light/dark cycle of 12 h light-12 h dark, temperature 25 ± 2 °C and food *ad libitum*. Females in estrus were individually housed overnight in the presence of a sexually experienced male. On the next day, vaginal plugs were checked to confirm successful mating; females were separated and singly housed till birth.

In experiment I, newborn mice were sorted by sex and euthanized at post-natal day (PND) 0, 2, 4, 6, 8 and 10 (n between 3 and 6). In experiment II, newborn mice were sorted by sex, at PND 0, females were injected subcutaneously either with 100 μg of testosterone propionate (Females TP) or with corn oil as vehicle (Females Veh) and males were injected with corn oil (Males Veh). Males Veh, females Veh and females TP were euthanized at PND 1, 2, 3 and 4.

At each time point, mice were euthanized by rapid decapitation, brains were extracted and the midbrain, the brainstem, the cerebellum and the olfactory bulbs were removed. Then the entire cerebral cortex (CC) from both hemispheres was dissected from the rest of the prosencephalon.

### Mitochondria isolation

Cortical mitochondrial fractions were obtained from tissue homogenates by differential centrifugation. Briefly, tissue was homogenized by twelve strokes with a glass-Teflon homogenizer in Tris-HCl 10 mM pH 7.4, containing 70 mM sucrose, 230 mM Mannitol and 1 mM EDTA and centrifuged at 700 g for 10 min to discard nuclei and cell debris. Then, the supernatant was centrifuged at 8000 g for 10 min and the enriched mitochondria pellet was resuspended in a minimum volume (20 μL) of the same buffer. The whole procedure was carried out at 4 °C.

### Mitochondrial cardiolipin isolation and fatty acid composition

Total lipids from an aliquot of mitochondrial fraction containing 2 mg of protein were extracted by the method of Folch^85^. Phospholipids were isolated by 2-D high-performance thin layer chromatography (HPTLC) on pre-coated silica gel plates (10 × 20 cm) from Whatman Schleicher and Schuell (Maidstone, England). The mobile phase for the first-D was chloroform:methanol:water:amonium hidroxide (65:25:4:0.5, v/v) and the mobile phase for the second-D was chloroform:acetone:methanol:acetic acid:water (36:48:12:12:6, v/v). Spots were visualized by iodine vapor. CL was identified by comparison with commercial standards and quantified by densitometric analysis using the Image J sofware (NIH). CL spots were scrapped off from the plate and extracted from the silica with chloroform:methanol (1:2, v/v). The extracts were dried under N_2_, and saponified with 10% KOH in ethanol (30 min at 85 °C). Unsaponified compounds were extracted with hexane and later, the aqueous phase acidified with 37% HCl (v/v) and extracted twice with hexane. Fatty-acid methyl esters (FAMEs) were synthesized by incubation for 1 hr at 85 °C in 10% BF_3_ in methanol. The resulting FAMEs were extracted with hexane and quantified by Gas-liquid chromatography using a capillary column (Omegawax 250) mounted on a Hewlett Packard HP 6890 Series GC System Plus (Avondale, PA). The FAMEs were identified by comparison of their relative retention times with authentic standards and mass distribution was calculated by quantification of the peak areas and expressed as relative quantity of each FAME from the total FAMEs in the sample. The saturation ratio was calculated with the relative amount of the identified saturated and polyunsaturated FAs (Saturation ratio = ∑ Saturated (16:0 + 18:0)/ ∑ Polyunsaturated (18:2 + 18:3 + 20:4 + 22:5 + 22:6)).

### Quantitative RT-PCR

Frozen tissue was homogenized in TRI Reagent^®^ Solution (Ambion), RNA isolation was performed according to manufacturer instructions. First-strand cDNA was prepared from 2 μg RNA using M-MLV reverse transcriptase (Promega, Madison, WI, USA). After reverse transcription, 5 μL of cDNA were amplified by real-time PCR in a 15 μL volume reaction using SYBR Green Mix (Applied Biosystems, Foster City, CA, USA) in the ABI Prism 7500 Sequence Detection System (Applied Biosystems) with conventional Applied Biosystems cycling parameters (40 cycles of changing temperatures, first at 95 °C for 15 seconds and then at 60 °C for a minute).

All the primer sequences (Table 1) were designed using Primer Express software (Applied Biosystems). For each primer pair an appropriate dilution of cDNA was chosen in order to achieve the same amplification efficiency than the housekeeping genes (18S rRNA, β-Actin and Rpl13A). Changes in mRNA expression were calculated using the DDCt method^86^, using the Best Keeper index^87^ as total mRNA load reference for each sample.

### Protein measurement

Protein content was determined by the method of Lowry^88^ using bovine serum albumin as standard.

### Statistical analysis

Data shown in the figures are presented as the mean ± standard error of the mean (SEM). The size of the experimental groups is indicated in each figure legend. Gaussian distribution of data sets was assessed by Kolmogorov-Smirnov test. Statistical significance was evaluated by two-way analysis of variance (ANOVA) followed by Bonferroni or Games-Howell *post*-*hoc* tests (depending on whether variances were homogeneous or not, respectively) for multiple comparisons. In those cases in which an interaction between two factors was not detected, data were split and each factor was analyzed by one-way ANOVA.

For the correlation studies Spearman’s test was applied to detect the association (not necessarily linear) between two continuous variables^89^.

SPSS v23.0 software (IBM Corp. Armonk, NY, USA) was used for the analysis. The significance level was set at p < 0.05.

## Additional Information

**How to cite this article:** Acaz-Fonseca, E. *et al*. Developmental Sex Differences in the Metabolism of Cardiolipin in Mouse Cerebral Cortex Mitochondria. *Sci. Rep.* **7**, 43878; doi: 10.1038/srep43878 (2017).

**Publisher's note:** Springer Nature remains neutral with regard to jurisdictional claims in published maps and institutional affiliations.

## Figures and Tables

**Table 1:** Primer sequence for quantitative-PCR.

Gene	Forward 5′–3′	Reverse 5′–3′
18 S RNA	CGCCGCTAGAGGTGAAATTCT	CATTCTTGGCAAATGCTTTCG
β-Actin	CAACTTGATGTATGAAGGCTTTTGGT	ACTTTTATTGGTCTCAAGTCAGTGTACAG
Rpl13A	TACCAGAAAGTTTGCTTACCTGGG	TGCCTGTTTCCGTAACCTCAAG
TAZ	CCTGAAGTTGATGCGTTGGA	GACACACAGGCACACATTTGC
CLS	TGTAATGTTGATCGCTGCTGTGT	CCTAGCCGTGGCATAGCAA
PGS-1	GCCAGGACCGCTACGTGTT	GCCCTGCAACTGCAAGGATA
iPLA2-γ	GGAATAGAAGTGAAGCACATTGCA	TAAGTCCCTTGGGAGCAGAAGT
LCLAT-1	GCATTTGTTAGTGGGAGAGTGCTA	GTAAGTTCCCAGCAGGATTAAAGTG
UCP-2	ACAAGACCATTGCACGAGAG	ATGAGGTTGGCTTTCAGGAG
PPAR-α	CAGCGTCACGTCTTGACCAA	CCCTCTACATAGAACTGCAAGGTTT
FASD-1	GGAATCACCTGCTACATCATTTTG	AGCAGTTAGGCTTGGCATGGT
FASD-2	GCCTGGTTCATCCTCTCGTACT	TGTTGCAGCCATCCAGCTT
